# Outstanding chain-extension effect and high UV resistance of polybutylene succinate containing amino-acid-modified layered double hydroxides

**DOI:** 10.3762/bjnano.10.68

**Published:** 2019-03-12

**Authors:** Adam A Marek, Vincent Verney, Christine Taviot-Gueho, Grazia Totaro, Laura Sisti, Annamaria Celli, Fabrice Leroux

**Affiliations:** 1 Institut de Chimie de Clermont-Ferrand (ICCF), SIGMA Clermont, CNRS, Université Clermont Auvergne, F-63000 Clermont-Ferrand, France; 2 Department of Organic Chemical Technology and Petrochemistry, Silesian University of Technology, 44100 Gliwice, Poland; 3 Dipartimento di Ingegneria Civile, Chimica, Ambientale e dei Materiali, Università di Bologna, Via Terracini 28, 40131 Bologna, Italy

**Keywords:** amino acid, layered double hydroxide, phenylalanine, polybutylene succinate

## Abstract

Polybutylene succinate (PBS) nanocomposite materials were prepared using a melt compounding process. The Mg_2_Al-based PBS nanocomposites, dispersed with inorganic–organic hybrid materials (layered double hydroxides, LDHs), were functionalized with the amino acids L-histidine (HIS) and L-phenylalanine (PHE). The rheological and anti-ultraviolet (anti-UV) properties were investigated and compared to filler-free PBS as well as LDH Mg_2_Al/nitrate as references. Both organo-modified LDHs exhibited a remarkable chain-extension effect for PBS with an outstanding increase in the zero-shear viscosity η_0_ for PBS–Mg_2_Al/PHE (two order of magnitude increase as compared to filler-free PBS). These results were compared to data found in the literature. Moreover, HIS and PHE anions embedded into the LDH structure can successfully prevent the chain scission reactions that usually occur during photo-ageing of PBS under UV radiation exposure. This highlights the outstanding performance of the LDH hybrid materials, and in particular, their application as a polymer chain extender and UV stabilizer for PBS, which can likely be extended to other biodegradable polymers.

## Introduction

Polymer nanocomposite materials have been one of the leading scientific topics over the past decades [1–4]. Because many petrochemical sources are non-renewable and require raw materials for large-volume polymer production, many studies have been focused on the development of their substitutes. One of the most promising candidates to replace petroleum-based polymers is polybutylene succinate (PBS) – a biodegradable aliphatic polyester, obtained from the renewable sources succinic acid and butane-1,4-diol via a polycondensation process. Indeed PBS has mechanical characteristics similar to well-known polyolefins, such as low-density poly(ethylene) (LDPE). However, the drastic drawback of PBS for possible use in everyday life (e.g., packaging) is its rapid hydrolysis and UV degradation [5].

A classical approach to overcome these problems is to add a stabilizing agent that may act as an anti-moisture and UV stabilizer. The small organic molecules used [6–9] are typically prone to migrate out of the polymer, thus creating some porosity and subsequently causing potential disruption in the polymer barrier integrity. In addition to this, the possible release of the stabilizers is a key issue in terms of health since they may come in contact with the nutrient product. It is of great importance to avoid the migration of such chemical additives.

Today inorganic containers are thought to play the dual role of embedding a specific agent to avoid its ingress into a polymer as well as providing the complementary properties as a gas barrier and mechanical reinforcement for the polymer. Among the candidates, layered double hydroxides (LDH) appear to be a promising choice in endowing multiple properties to the polymer. This due to their versatility in terms of chemical composition and the relatively straightforward preparation involving soft chemistry routes.

Layered double hydroxides can be obtained as naturally existing materials or can be produced by synthetic routes (e.g., co-precipitation or ion exchange). They are also known as anionic clays or hydrotalcite-like materials and are described by the general formula [M^II^_1−_*_x_*M^III^*_x_*–(OH)_2_](A*^n^*^−^)*_x_*_/_*_n_*·*m*H_2_O, where M^II^ and M^III^ are di- and trivalent cations respectively and A*^n^*^−^ is an anion. The presence of trivalent cations results in a positive charge on the layers, which has to be balanced by diverse inorganic or organic anions intercalated between the layers [10–11]. LDHs are considered to be “green” and low environmental impact fillers [12], biocompatible [13] and food compatible [14].

Indeed the choice of organo-modified LDH using amino acids, and among them the protein-building amino acids, has already been demonstrated [15]. Having in mind that UV stabilization should require UV absorption properties for the organic molecule, any proteinogenic amino acids presenting cycle in their backbone will be preferentially considered. Tyrosine and tryptophan have been studied for their UV stabilizing properties; however, histidine (HIS) and (PHE) are rarely reported. One study reports the beneficial role of PHE interleaved into LDH as a self-healing agent for polymer coating in corrosion inhibition for aluminium substrates [16]. Due to the presence of benzene (for PHE) or the heterocyclic imidazole ring (for HIS), both organic molecules should absorb in the short to medium wavelength range of the ultraviolet C (UVC) and ultraviolet B (UVB) regions.

In the present work, Mg_2_Al LDH materials were first organo-modified with histidine and phenylalanine using a co-precipitation method and then characterized by X-ray diffraction (XRD), Fourier transform infrared spectroscopy (FTIR) and thermogravimetric analysis (TGA). As expected, the UV–vis spectra showed absorbance in the UVC and UVB regions and the potential role as UV stabilizers. In a second part, PBS composites were prepared with 5 wt % of LDH filler by melt blending and once again fully characterized (XRD, TGA, DSC, DMTA and melt rheology). Impressively a pronounced chain-extension effect for PBS was observed with both organo-modified LDHs, especially in the case of PBS–LDH/PHE, for which the apparent molecular weight was almost 400 times higher than the pristine PBS. Finally, PBS composites were subjected to a photodegradation process, showing their resistance to UV irradiation.

## Experimental

### Materials and reagents

Aluminium nitrate Al(NO_3_)_3_·9H_2_O, magnesium nitrate Mg(NO_3_)_2_·6H_2_O, sodium nitrate NaNO_3_, sodium hydroxide, the amino acids L-histidine (HIS) and L-phenylalanine (PHE) were purchased from Sigma-Aldrich. PBS (PBE003) was purchased from NaturePlast. All the materials were reagent grade and used as received.

### Layered double hydroxide synthesis

The LDH hybrids with general formula Mg_2_Al(OH)_6_[amino acid]·2H_2_O were prepared by a co-precipitation method according to a similar procedure as described by Totaro et al. [17], for possible comparison. The solution of Mg(NO_3_)_2_·6H_2_O and Al(NO_3_)_3_·9H_2_O (molar ratio 2/1) in milli-Q water (100 mL) was added dropwise to the vigorously stirred water solution of organic guest (50 mL) for 3.5 h and the pH was kept constant (10.0 ± 0.1) with the addition of NaOH solution. When the addition of salts was finished, the reaction mixture was aged for 3.5 h at room temperature. Both the reaction and ageing processes were performed under nitrogen atmosphere to avoid contamination by carbonate. The final products were centrifuged, washed several times with deionized water and vacuum dried at 40 °C for 24 h. Next, the solid LDH fillers were ground using a Retsch CryoMill machine and sieved to obtain a fraction with particles of diameter less than 50 µm.

### Melt blending and film making

The PBS–LDH nanocomposites were obtained using a twin screw extruder Hakke MINILAB microcompounder (Thermo Electron Corporation). The melt extrusion process was performed at 120 °C with a roller speed of 100 rpm over 5 min. A LDH mass loading of 5 wt % was used as it appeared to be the right compromise to be detected by XRD, as formerly demonstrated [15]. This was also shown to endow the polymer composites with significantly improved properties as well as to envision a master-batch considering the observed state of dispersion.

PBS–LDH nanocomposite films with thickness of about 90–100 µm were prepared by compression moulding between two Teflon sheets under 100 bar at 120 °C for one minute.

### Photodegradation

PBS and PBS–LDH nanocomposite films were placed on a rotating carousel in a SEPAP 12/24 chamber from ATLAS equipped with four 400 W mercury lamps with spectral rays above 300 nm. The accelerated photodegradation was studied at 60 °C and under air atmosphere.

### Characterization methods

FTIR spectra of the LDH fillers were recorded using a Nicolet 380 FTIR spectrometer (DTGS detector) equipped with an attenuated total reflection single reflection diamond from Specac, and 32 scans and at a resolution of 4 cm^−1^ were collected.

LDH fillers and PBS–LDH nanocomposites were characterized by XRD using a Philips X-Pert Pro diffractometer with Cu Kα radiation. Data were collected at room temperature from 2θ = 2.0–70.0° with a step size of 0.03° and a counting time of 10 s per step.

UV–vis spectra of the LDH substances and polymer nanocomposites were obtained using a Shimadzu UV-2101 PC spectrophotometer. Kubelka–Munk theory was applied to the LDH filler data to transform the diffuse reflectance spectra into absorption spectra.

The fluorescence spectra of PBS–LDH nanocomposites were recorded with a Perkin-Elmer LS-55 luminescence spectrophotometer equipped with a front surface accessory and pulsed xenon excitation source. The emission signal was collected with the monochromater from 200 to 600 nm at scan rate of 600 nm min^−1^ and excitation wavelength of 280 nm.

The thermal properties of the LDH fillers and polymer composites were characterized by differential scanning calorimetry (DSC) using a Perkin-Elmer DSC6 apparatus. The analyses were carried out under nitrogen, and firstly, the samples (≈10 mg) were heated from 40–150 °C at 20 °C min^−1^, then kept at high temperature for 2 min, and then cooled down to −60 °C at 10 °C min^−1^. After that, the thermal history of the samples was deleted and a second scan profile was performed by heating from −60 °C to 150 °C at 10 °C min^−1^. The glass transition temperature (*T*_g_), the melting temperature (*T*_m_) and the enthalpy of fusion (∆*H*_m_) were measured from the second scan. *T*_g_ was taken as the midpoint of the heat capacity increment associated with the glass-to-rubber transition. The crystallization temperature (*T*_c_) and the enthalpy of crystallization (∆*H*_c_) were measured during the cooling scan.

Thermogravimetric analysis (TGA) of LDH fillers and composites was performed in air atmosphere (gas flow 30 mL min^−1^) using a Perkin Elmer TGA7 apparatus. The temperature range of 50–850 °C and heating rate 10 °C min^−1^ was applied. The onset degradation temperature (*T*_onset_) was taken from the intersection of the tangent of the initial point and the inflection points. The 10% mass loss temperature (*T*_10_^D^) was also measured.

The melt rheological properties of the polymer composites were measured at 120 °C using a dynamic mechanical spectrometer (ARES Rheometric Scientific T&A Instruments) equipped with two parallel plate holders of 8 mm in diameter. The measurements were performed in oscillatory frequency sweep mode with the range of frequency sweeps from 0.1 to 100 rad s^−1^ and the gap between plates set at 1 mm. In all cases, the oscillatory shear stress amplitude was checked to ensure that measurements were performed inside the linear viscoelastic domain. The storage modulus (*G*’), loss modulus (*G*”) and tan δ (ratio of *G*” and *G*’) were monitored automatically against frequency.

The dynamic mechanical thermal properties were measured using a Rheometric Scientific DMTA IV dynamic mechanic thermoanalysis (DMTA) instrument with a dual cantilever testing geometry. Test samples were prepared by injection moulding at 140 °C using a Minimix Molder, obtaining small-sized bars (33 × 8 × 2 mm). Such samples were scanned from −150 °C to 80 °C (heating rate 3 °C min^−1^, frequency 3 Hz, strain 0.01%).

## Results and Discussion

### Layered double hydroxide filler characterization

The organo-modified Mg_2_Al LDH hybrid materials containing the levorotary form of amino acids: L-histidine (HIS) and L-phenylalanine (PHE) intercalated between metal cation layers were prepared by the co-precipitation method.

The synthesis and characterization of LDH–phenylalanine compounds has already been reported by Aisawa et al. [18]. However, we propose here a different interpretation of the XRD patterns based on a more detailed analysis of the position of the X-ray diffraction peaks and the matching between the surface area per unit charge of LDH host and the organic guest.

**Figure 1:**
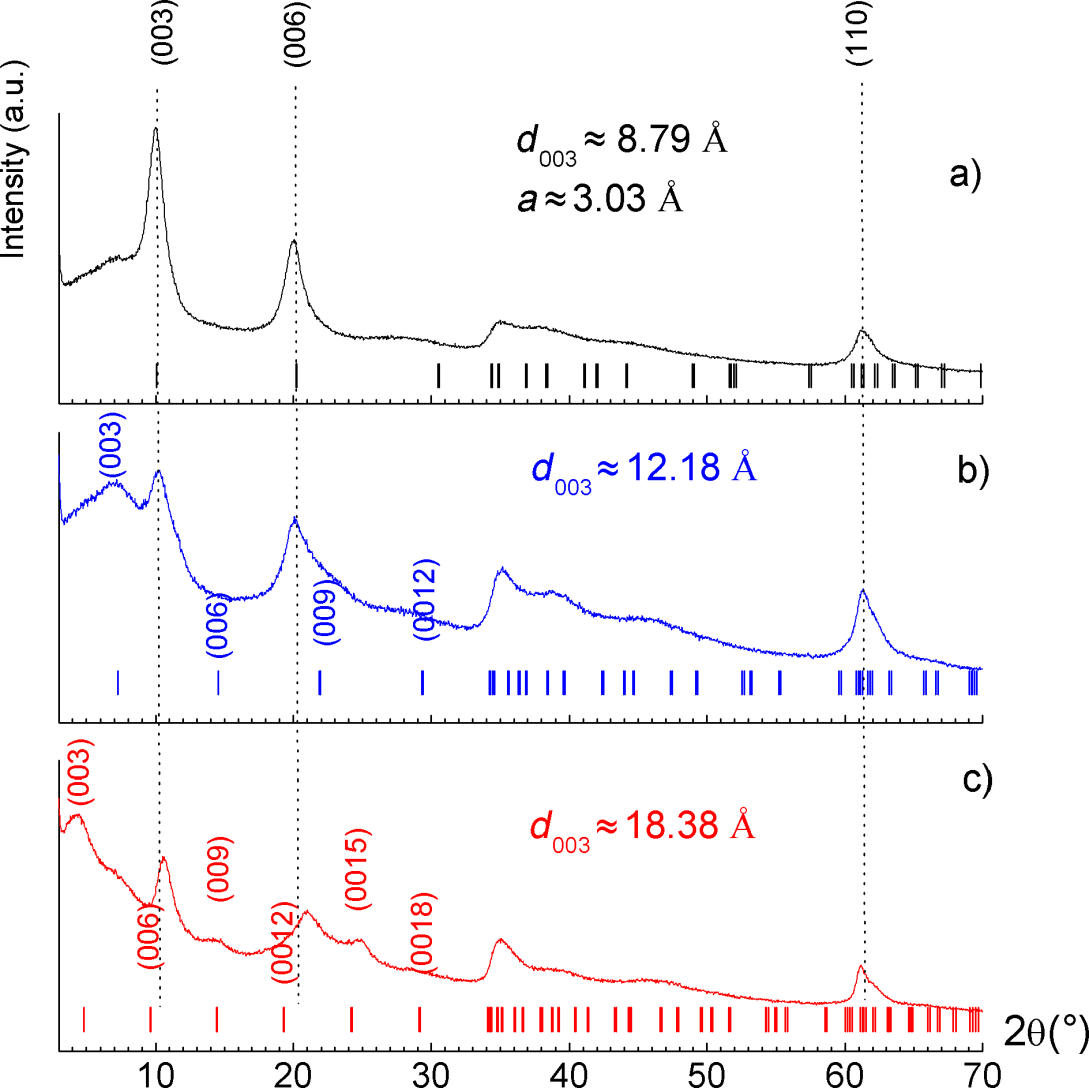
XRD patterns of Mg_2_Al hybrids: a) LDH/nitrate, b) LDH/HIS and c) LDH/PHE. The ticks under the peaks give the positions of the Bragg reflections assuming 3R polytypes.

Mg_2_Al/nitrate, as a reference sample, displays an X-ray diffraction pattern typical of lamellar compounds with a series of strong basal 00*l* reflections at low angles (Figure 1a). In the present case, the reflections are indexed assuming a three-layer 3R polytype with rhombohedral symmetry (space group *R*3*m*), generally observed in LDH systems. The interlayer distance can be determined in a straightforward way from the position of the first (00*l*) reflection indexed as (003), leading here to a value of ≈8.79 Å as expected for a nitrate-containing LDH. The position of the (110) reflection at high angles, near 2θ = 60° for Cu Kα radiation, allows the value of the lattice parameter *a* to be determined since *a* = 2*d*_110_. Its value reflects the radii of the cations and the M^2+^/M^3+^ ratio within the hydroxide layers. The value obtained here is ≈3.03 Å and suggests a Mg^2+^/Al^3+^ molar ratio slightly lower than 2 [19].

**Figure 2:**
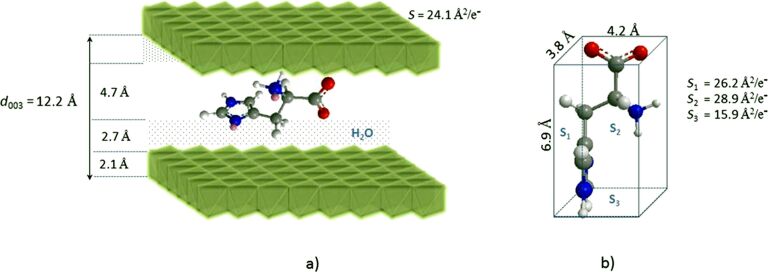
a) Structural model for Mg_2_Al/HIS, b) molecular structure of L-histidine, dimensions and corresponding surface area per unit charge calculated by considering a rectangular parallelepiped shape.

For the synthesis performed in the presence of histidine and phenylalanine, we observed two series of (00*l*) reflections: one attributed to the desired Mg_2_Al LDH hybrid phase and the other to a LDH/nitrate phase. In Figure 1, the Miller indices (*hkl*) were assigned to each reflection (assuming 3R polytypes) with positions marked by ticks under the peaks. The presence of this nitrate phase, also evidenced by FTIR analysis (see below), can be explained by either the small excess of the organic anion used during the synthesis or the large excess of nitrate anions in the reaction medium introduced by the reactants in the form of nitrate salts. The diffraction pattern obtained for LDH/HIS is poorly defined with only one (00*l*) reflection visible, from which the interlayer distance was estimated as ≈12.2 Å (Figure 1b). In the case of LDH/PHE, additional (00*l*) reflections are observed and their positions are consistent with an interlayer distance of ≈18.4 Å (Figure 1c). By subtracting both the thickness of the LDH hydroxide layer ≈2.1 Å and the hydrogen bond distances ≈2.7 Å from these interlayer distances, one can estimate the space available for the organic anion along the *c* direction. A value of ≈4.7 Å was thus obtained for LDH/HIS and ≈10.9 Å for LDH/PHE. A comparison with the dimensions of the organic anions (determined using ChemBio 3D ultra 13.0 suite software) leads us to propose a monolayer arrangement for LDH/HIS where the main plane of the molecule is oriented nearly parallel to the hydroxide layer (Figure 2). From the point of charge density, Mg_2_Al hydroxide layers display an available surface area per unit charge of ≈24 Å^2^, a value close to that required by histidine in a parallel orientation (≈26–29 Å^2^/e^−^). This interlayer arrangement suggests an important confinement of histidine molecules between LDH layers. This may explain the presence of co-intercalated nitrate ions, which decreases the constrained accommodation that would have resulted in the close proximity of the HIS molecules to one other.

**Figure 3:**
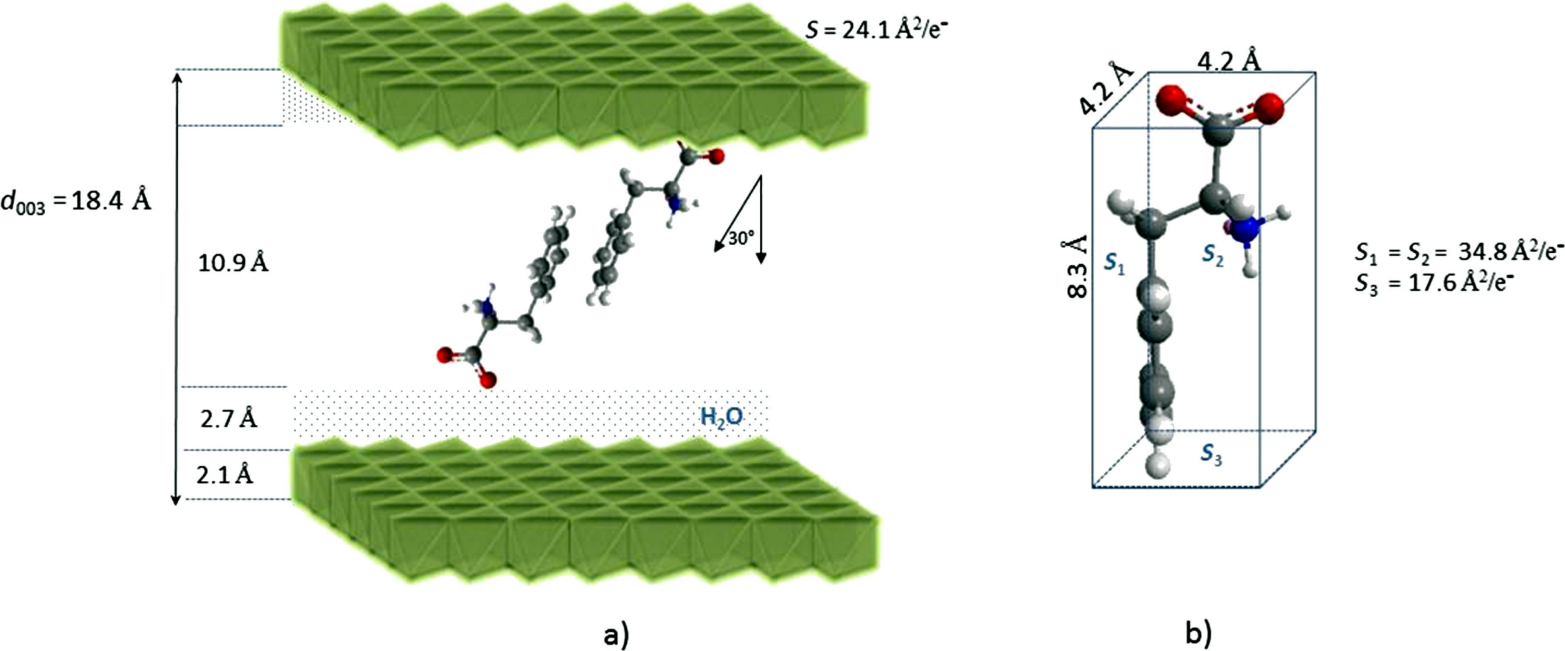
a) Structural model for Mg_2_Al/PHE, b) molecular structure of L-phenylalanine, dimensions and surface area per unit charge calculated by considering a rectangular parallelepiped shape.

On the other hand, the interlayer distance in the case of LDH/PHE must arise from a bilayer head-to-tail arrangement in the direction perpendicular to the hydroxide layer (Figure 3). A parallel orientation similar to histidine would require a surface area per unit charge of ≈35 Å^2^, which is too high compared to the charge density of Mg_2_Al host layers, thus supporting the perpendicular orientation. Actually, phenylalanine molecules are likely to be oriented in an inclined manner and partially interpenetrating at the aromatic rings, allowing for the structurally beneficial π–π interaction.

**Figure 4:**
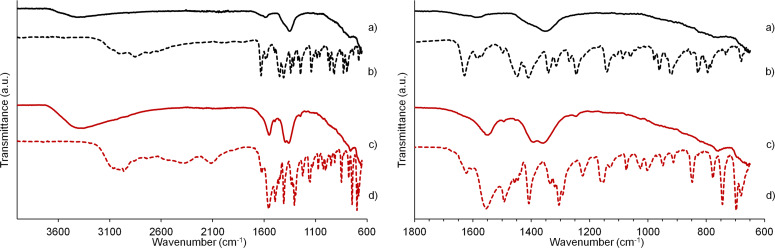
FTIR spectra of organo-modified Mg_2_Al hybrids: (a) LDH/HIS, (c) LDH/PHE and pristine amino acids: (b) L-HIS, (d) L-PHE (left side), zoom region on the right side.

FTIR spectroscopy was applied to confirm the intercalation of HIS and PHE in the hybrid structures. The FTIR spectra of LDH/HIS and LDH/PHE in comparison to pristine amino acids are displayed in Figure 4. The large, broad bands with maximum around 3400 cm^−1^ correspond to the O–H stretching vibrations of the hydroxide layers and water molecules located in the interlayered spaces or physically adsorbed on the surface of hybrids. The typical signals from amide functional groups (N–H, C=O, C–N) overlap in the range of 1700–1480 cm^−1^ and the lattice vibration of M–O in the platelet structure can be depicted at a low wavenumber (800–650 cm^−1^).

The experimental chemical composition of all synthesized LDHs has been estimated based on TGA analysis and collected in Table 1. All the thermogravimetric curves are shown in the Supporting Information File 1, Figure S1. The thermal decomposition of LDH occurs as a multiple step process. Firstly, the interlayer and surface adsorbed water molecules are removed up to around 200 °C. The weight change from 200–400 °C can be attributed to the removal of hydroxyl group OH^−^ associated with metal cations Al^3+^ and Mg^2+^. Finally, the third weigh loss at >400 °C can be observed for the decomposition of amino acid or nitrate anions and the residual mass at 800 °C can be considered as the mass residue of Mg_2_AlO_7/2_. Evidently these residual mass losses are not equal because of the difference in molecular weight of the guest organic species as well as the different hydration rate.

**Table 1:** Experimental chemical compositions of Mg_2_Al LDHs.

Code	Experimental chemical composition^a^

LDH/nitrate	[Mg_2_Al(OH)_6_](NO_3_^−^)_1.0_·0.75H_2_O
LDH/HIS	[Mg_2_Al(OH)_6_](HIS^−^)_0.35_(NO_3_^–^)_0.65_·1.14H_2_O
LDH/PHE	[Mg_2_Al(OH)_6_](PHE^−^)_0.65_(NO_3_^–^)_0.35_·2.21H_2_O

^a^Anions and water molecule content determined by TGA under air flow. Mass formulae are: 253.04 g mol^−1^ for Mg_2_Al/nitrate, 346.26 g mol^−1^ for Mg_2_Al/PHE and 292.4 g mol^−1^ for Mg_2_Al/HIS. The procedure and detailed calculations are presented in Supporting Information File 1 and the TGA traces are displayed in Figure S1.

**Figure 5:**
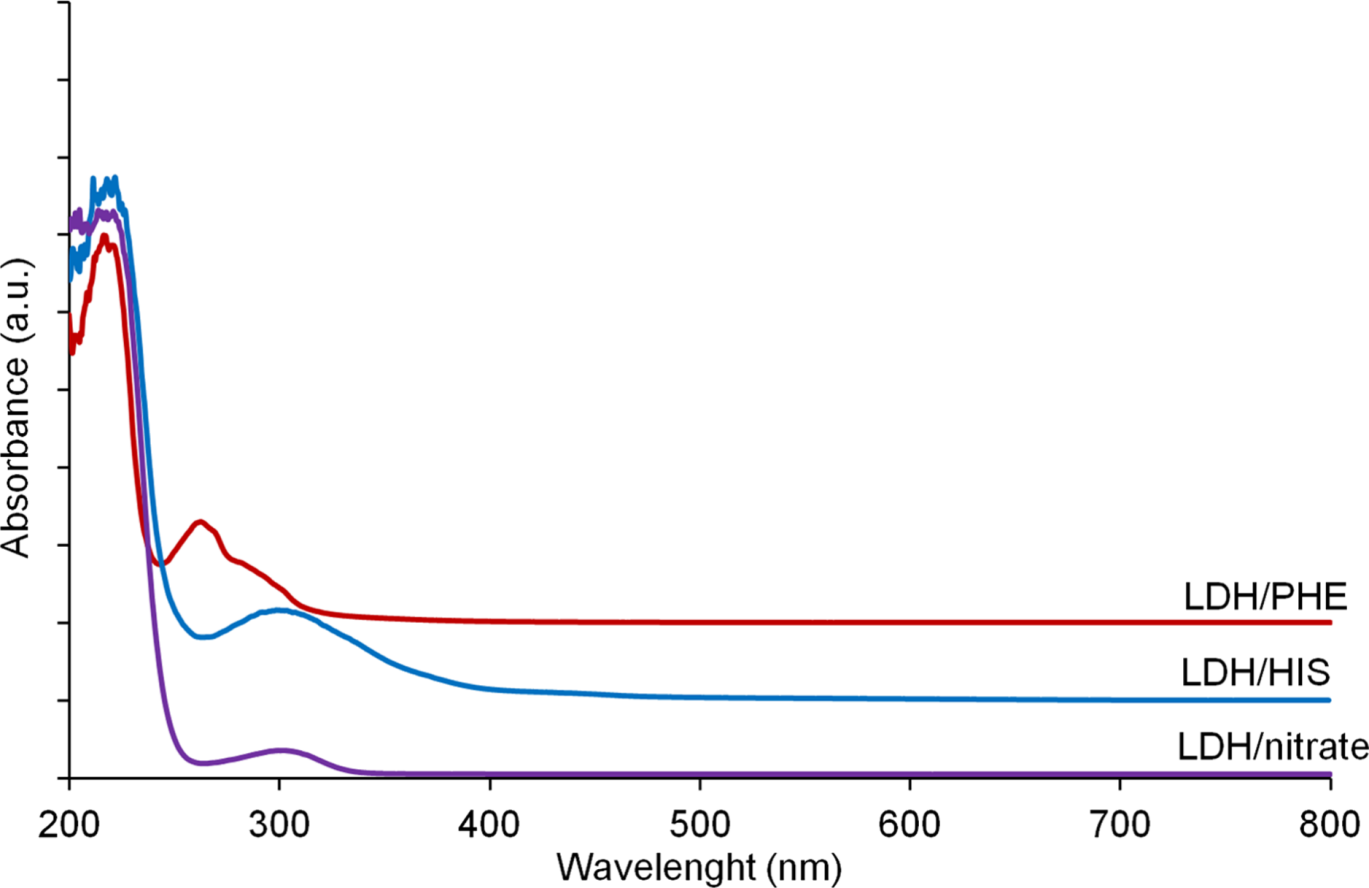
Diffuse reflectance UV–vis spectra (Kubelka–Munk functions) of Mg_2_Al LDHs.

The UV-absorbing properties of the LDH structure with amino acids are presented in Figure 5. The nitrate–inorganic derivative, which is used as a reference, presents a typical quite low absorption with a maximum at about 302 nm, coming from the presence of nitrate anions in the interlayer space [20]. When PHE is inserted into the LDH inorganic structure, the absorption in the UV domain increases and a hypsochromic shift is observed with a maximum at 262 nm. In the case of LDH/HIS, the absorption is also more intense than for the nitrate reference, but the maximum shifts to 298 nm. As surmised, both cyclic amino acid organo-modified Mg_2_Al LDHs should be effective as potential UV stabilizers.

### PBS–LDH filler composites – characterization

**Figure 6:**
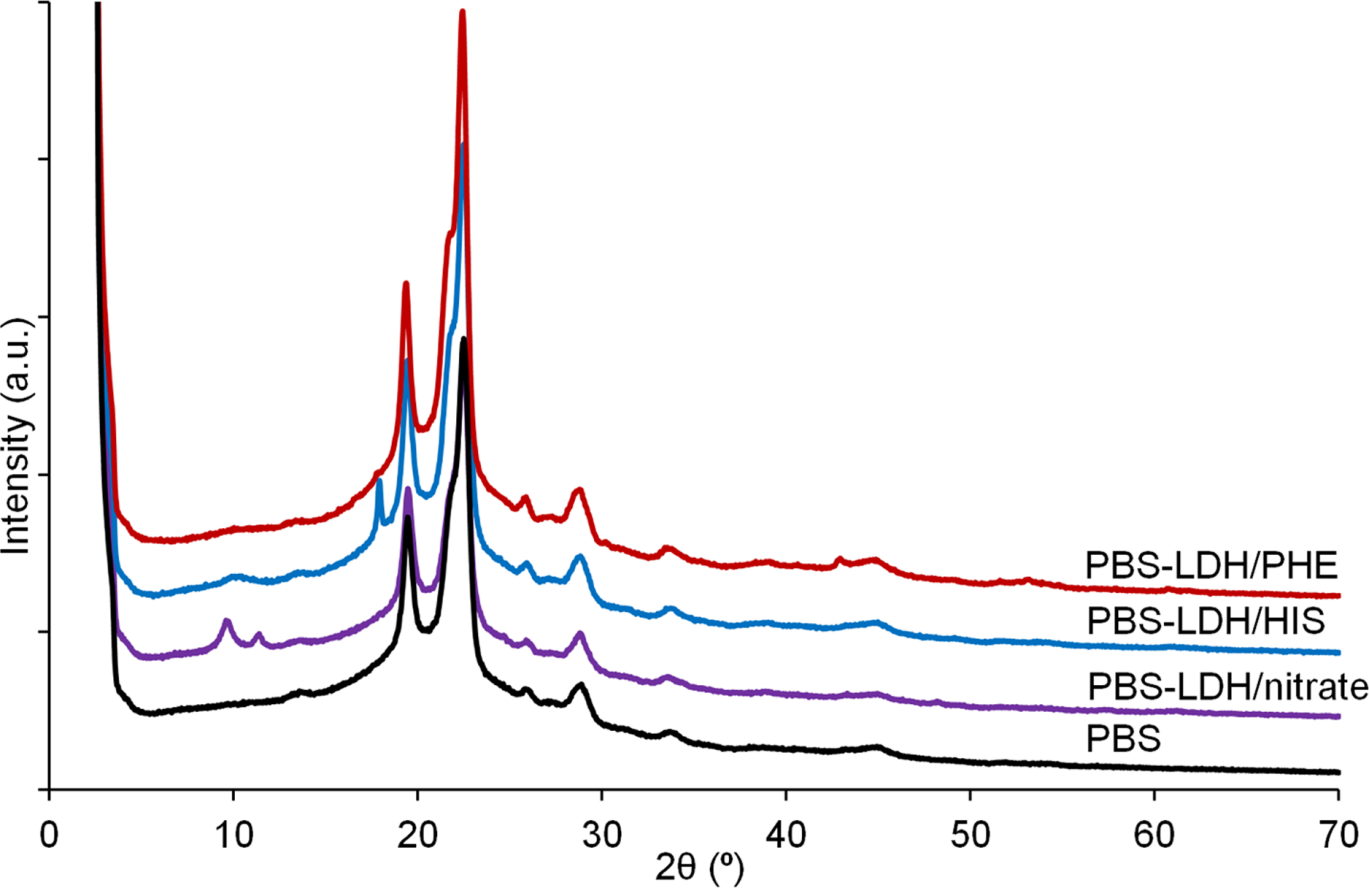
XRD patterns of PBS nanocomposites with 5 wt % Mg_2_Al LDH fillers.

PBS nanocomposites with 5 wt % of amino-acid-modified Mg_2_Al LDHs and Mg_2_Al LDH nitrate were prepared by melt extrusion. The XRD patterns are presented in Figure 6 together with PBS without filler as a reference.

The diffraction peaks located at 2θ = 18–30°, which are characteristic for the crystallinity of PBS, seem to not be affected by the addition of the LDH hybrid materials. There is no reflection coming from pristine LDHs modified by amino acids. In contrast, the PBS–LDH nitrate composite exhibits an initial basal spacing of 0.84 nm. In the case of PBS composites with LDH/PHE or LDH/HIS, the absence of harmonic peaks from the layered filler structure can be explained by their lower crystallinity or by a partial exfoliation occurring during thermal processing. For PBS–LDH/HIS, there is an additional peak at 2θ = 18° that is difficult to assign. Its associated small full width at half maximum (FWHM) may be attributed to rather structurally well ordered organic species. Tentatively it may be ascribed to some degradative effect coming from the imidazole cycle since there is no such effect for the other PBS composites. It is well known that imidazole cycle is prone to coordinate to metal ions as in metallo-proteins [21]. The temperature used during the extrusion may have induced a reaction of HIS weakly tethered onto the LDH platelets surface. Another possibility could be some reaction with PBS chains. However, this can be excluded since the calorimetric properties of PBS are maintained (Table 2).

**Table 2:** Thermogravimetric and calorimetric data of PBS nanocomposites with 5 wt % of Mg_2_Al LDHs fillers.

Sample	*T*_onset_ (°C)^a^	*T*^10^_D_ (°C)^a^	Res mass (%)^a^	*T*_C_ (°C)^b^	∆*H*_C_ (J g^−1^)^b^	*T*_g_ (°C)^c^	*T*_m_ (°C)^c^	∆*H*_m_ (J g^−1^)^c^

PBS	385	362	0.0	85	63	−31	115	47
PBS–LDH/nitrate	360	327	3.3	85	58	−31	115	48
PBS–LDH/HIS	362	347	2.5	83	61	−29	115	53
PBS–LDH/PHE	366	345	1.9	82	55	−33	114	37

^a^Determined by TGA at 10 °C min^−1^ in air. ^b^Determined by DSC during the cooling scan from the melt at 10 °C min^−1^. ^c^Determined by DSC during the 2nd heating scan at 10 °C min^−1^.

The introduction of LDH fillers into PBS does not improve the thermal stability of the polymer and the decomposition process proceeds mainly in the range 300–450 °C. The determined *T*_onset_ and *T*^10^_D_ (temperature at which the polymer loses 10 wt %) measured for all nanocomposites are consistently lower than for PBS (Table 2). This trend has been previously observed and described in the literature [22–23]. It can be explained by the catalytic effect of Mg and Al ions on the intramolecular and intermolecular transesterifications of PBS and its hydrolysis due to water, which is released during the decomposition of the brucite-type layers. The calculated residual masses correspond with the filler loading. The addition of LDHs does not affect the melting temperature *T*_m_ which is quite constant at 115–114 °C. A slight decrease can be observed in the crystallization temperature *T*_c_ for nanocomposites with LDH/amino acids, from 85 °C (PBS) to 83 and 82 °C for PBS–LDH/HIS and PBS–LDH/PHE, respectively. Small differences are also observed in *T*_g_, but without a regular trend. However, *T*_g_ will be discussed further in the next paragraph, because DMTA is a more sensitive method to detect this. TGA and DSC traces for all nanocomposites are shown in the Supporting Information File 1, Figure S2 and Figure S3.

### Chain-extending effect and dynamic mechanical properties

The effect of synthesized amino acid LDH fillers on the chain extending and molecular weight evolution was measured using melt rheology. The rheological data were plotted in Cole–Cole plots – a model curve used to predict the variation in complex viscosity components, where the imaginary viscosity (η”) versus real viscosity (η’) is plotted as a circle arc in the complex plane. This representation is very helpful in the analysis of polymer and polymer composites [24–26]. By fitting and extrapolation of the Cole–Cole representation to the *x*-axis (η′ at η″ = 0), the Newtonian zero-shear viscosity η_0_ can be calculated using Equation 1, which reflects even small changes in molecular mass.

[1] $$\eta_{0}\propto M_{w}{}^{a}$$

**Figure 7:**
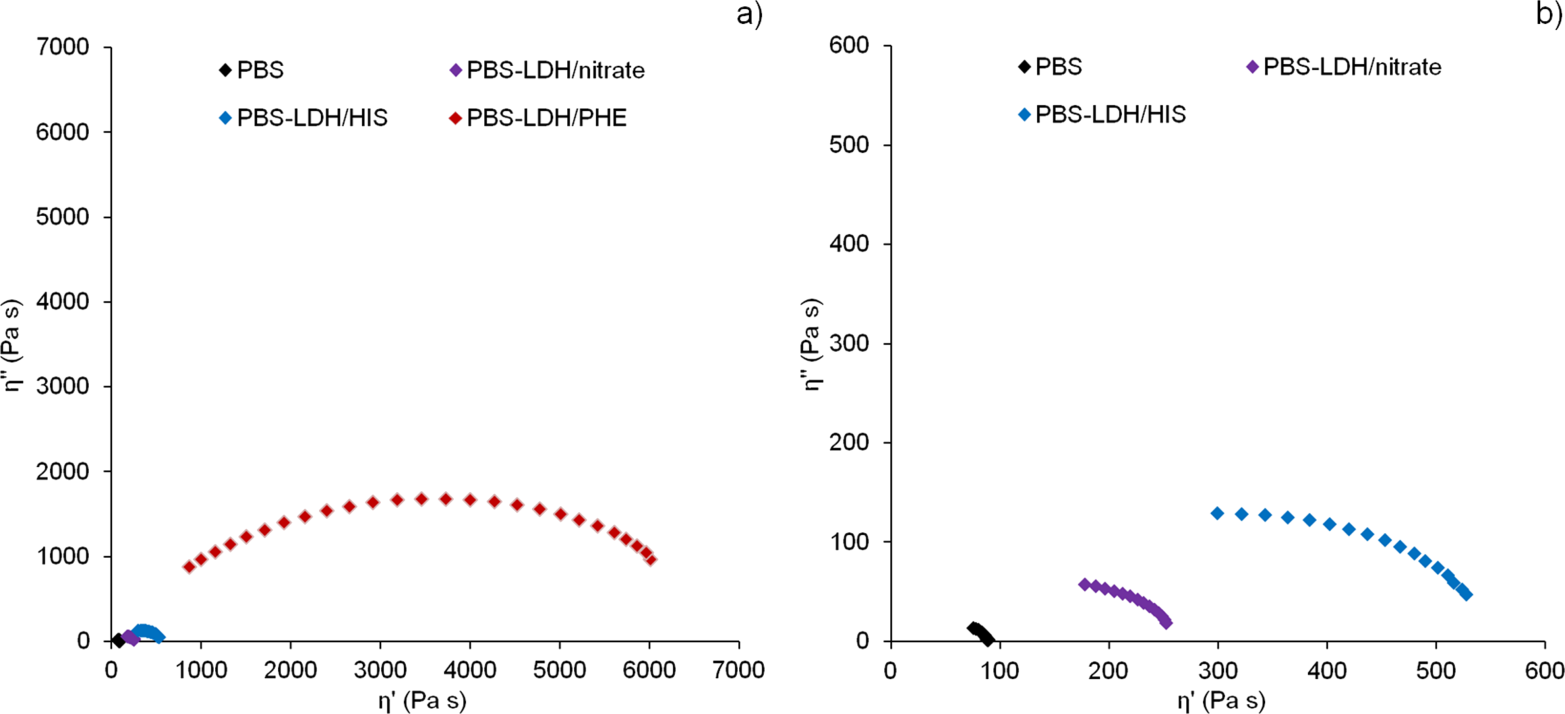
Cole–Cole plots of PBS and PBS nanocomposites with Mg_2_Al LDH (a), zoom region (b).

The effect of 5 wt % Mg_2_Al LDH filler on the PBS chain extension is presented as η”–η’ Cole–Cole plots in Figure 7. The addition of all LDH fillers causes the chain-extending effect associated with the non-miscible structure. The calculated values of the Newtonian zero-shear viscosity for PBS and nanocomposites with LDH/nitrate and LDH/HIS are 90, 274 and 584 Pa s. The best results were obtained for LDH/PHE with a calculated value of η_0_ of 7174 Pa s which is almost 80 times higher than for PBS.

The role of layered double hydroxides modified with 3-(4-hydroxyphenyl)propionic acid and amino acids such as tyrosine, tryptophan as PBS chain-extenders has been known and described previously, however the results were not so spectacular [15,17,23]. The significant effect of LDH/PHE most likely results from chemical interaction between tethered amino acid molecules and PBS chains. Counterintuitively, the imidazole ring is found to induce less chain extension ability than the non-polar benzene ring. Indeed the NH functional group that can conjugate is a capto-proton, therefore this function is not suitable to interact strongly with the carbonyl groups present along the aliphatic polyester. The absence in both cases of chemical affinity between the cyclic backbone and the polymer chains suggests that the organic molecules tethered to LDH platelets may have a more jammed effect as evidenced previously [27]. The powdered flake structure of LDH/PHE observed by scanning electron microscopy (SEM) (Supporting Information File 1, Figure S4) may also contribute to the strong interaction with PBS chains.

With a value of *a* = 3.4 in Equation 1, the ratio of apparent molecular weight between the composite with Mg_2_Al/PHE and filler-free PBS is of about 400 times (*M*_w_(PBS–Mg_2_Al/PHE)/*M*_w_(PBS) = 397, resulting in a similar change in the average repeat units under melt state polymer rheology), thus underlining once again the outstanding effect of the filler in networking PBS chains. This occurs without a transition to a gel-like structure, which would be deleterious for the PBS processability.

Table 3 reports quasi-exhaustive data related to the effect of organo-modified LDHs on the chain extension for PBS nanocomposites. Nano-hybrid fillers (based on Mg_2_Al or Zn_2_Al LDH cations and with different anions from carboxylic and dicarboxylic acids, ascorbic acid and amino acids) were incorporated to PBS polymer by both methods: in situ polymerization and melt extrusion. Depending on the type of organic molecules, chain extension or plasticizing effects were observed.

**Table 3:** Polymer processing and Newtonian zero-shear viscosity η_0_ for PBS–LDH nanocomposites.

Polymer processing	LDH cations	Interleaved anions^b^	Loading (wt %)	η_0_^a^ (Pa s)	Ref.

in situ polymerization	Mg_2_Al	succinate	3.0	250	[22]
	Mg_2_Al	sebacate	3.0	150	
	Mg_2_Al	adipate	3.0	–	
	Mg_2_Al	lauryl sulfate	3.0	–	
	Mg_2_Al	stearate	3.0	430	
	Zn_2_Al	stearate	3.0	–	
	Mg_2_Al	citric	3.0	55	
	Mg_2_Al	ricinoleic	3.0	180	

in situ polymerization	Mg_2_Al	PBS oligomer	3.0	1400	[28]
	Zn_2_Al	PBS oligomer	3.0	1000	

in situ polymerization	Mg_2_Al	HPP	3.0	400	[23]
	Zn_2_Al	HPP	3.0	790	

in situ polymerization	Mg_2_Al	HPP	2.5	331	[17]
	Mg_2_Al	ASA	2.5	255	
	Mg_2_Al	TRP	2.5	458	
	Mg_2_Al	TYR	2.5	243	

extrusion	Zn_2_Al	TRP	5.0	110	[15]
	Zn_2_Al	TYR	5.0	245	

extrusion	Zn_2_Al	cinnamic	5.0	360	[29]
	Zn_2_Al	*p*-hydroxycinnamic	5.0	120	
	Zn_2_Al	ferulic	5.0	190	
	Zn_2_Al	cafeic	5.0	170	

extrusion	Zn_2_Al	CH_3_(CH_2_)_6<_*_n_*_<16_COO^−^ (C8 to C18)	3.0	160 to 220	[30]

extrusion	Zn_2_Al	TYR	5.0	171*	[31]
	Zn_2_Al	TRP	5.0	179*	
	Zn_2_Al	HPP	5.0	138*	
	Zn_2_Al	NO_3_	5.0	76*	

extrusion	Mg_2_Al	HIS	5.0	584*	this article
	Mg_2_Al	PHE	5.0	7174*	
	Mg_2_Al	NO_3_	5.0	274*	

^a^η_0_ = 115 Pa s (*90 Pa s) for PBS by extrusion, η_0_ = 40–50 Pa s for PBS by in situ polymerization. ^b^HPP = 3-(4-hydroxyphenyl)propionic acid, ASA = L-ascorbic; TRP = L-tryptophan, TYR = L-tyrosine, NO_3_ = nitrate, HIS = L-histidine, PHE = L-phenylalanine.

In the case of in situ polymerization, an increase in the Newtonian zero-shear viscosity of nearly 20–28 times was observed when LDHs with embedded PBS oligomers were used, whereas the smaller molecules increased η_0_ from 40–50 Pa s for unmodified PBS 400, 430, 450 and 790 Pa s for Mg_2_Al/HPP, Mg_2_Al/stearate, Mg_2_Al/TRP and Zn_2_Al/HPP, respectively (when used at 2.5–3 wt %).

The effectiveness of the LDHs was lower when they were added to the PBS in the melt extrusion process, most likely because LDH fillers were less dispersed in this case compared to the in situ polymerization approach. The best results were obtained for Zn_2_Al/TYR and Zn_2_Al/cinnamic LDHs (5 wt %), from 115 to 245 and 360 Pa s respectively, while in other cases the differences were smaller. Our present results are here spectacular, since an increase of 80 times for η_0_ is observed, from 90 Pa s (unmodified PBS) up to more than 7,000 Pa s for Mg_2_Al/PHE LDH (5 wt %), respectively. Moreover, the PBS nanocomposite composition displays the desired UV-stabilizing effect (described below), which was described previously but only for PBS composites with Zn_2_Al/amino acid LDHs [15,31]. This work, however, used a different LDH platelet composition (Zn_2_Al) that is known to present some UV screening due to Zn^2+^ cations. However, hydrotalcite-type Mg_2_Al (as used in this work) has yet to be investigated.

**Figure 8:**
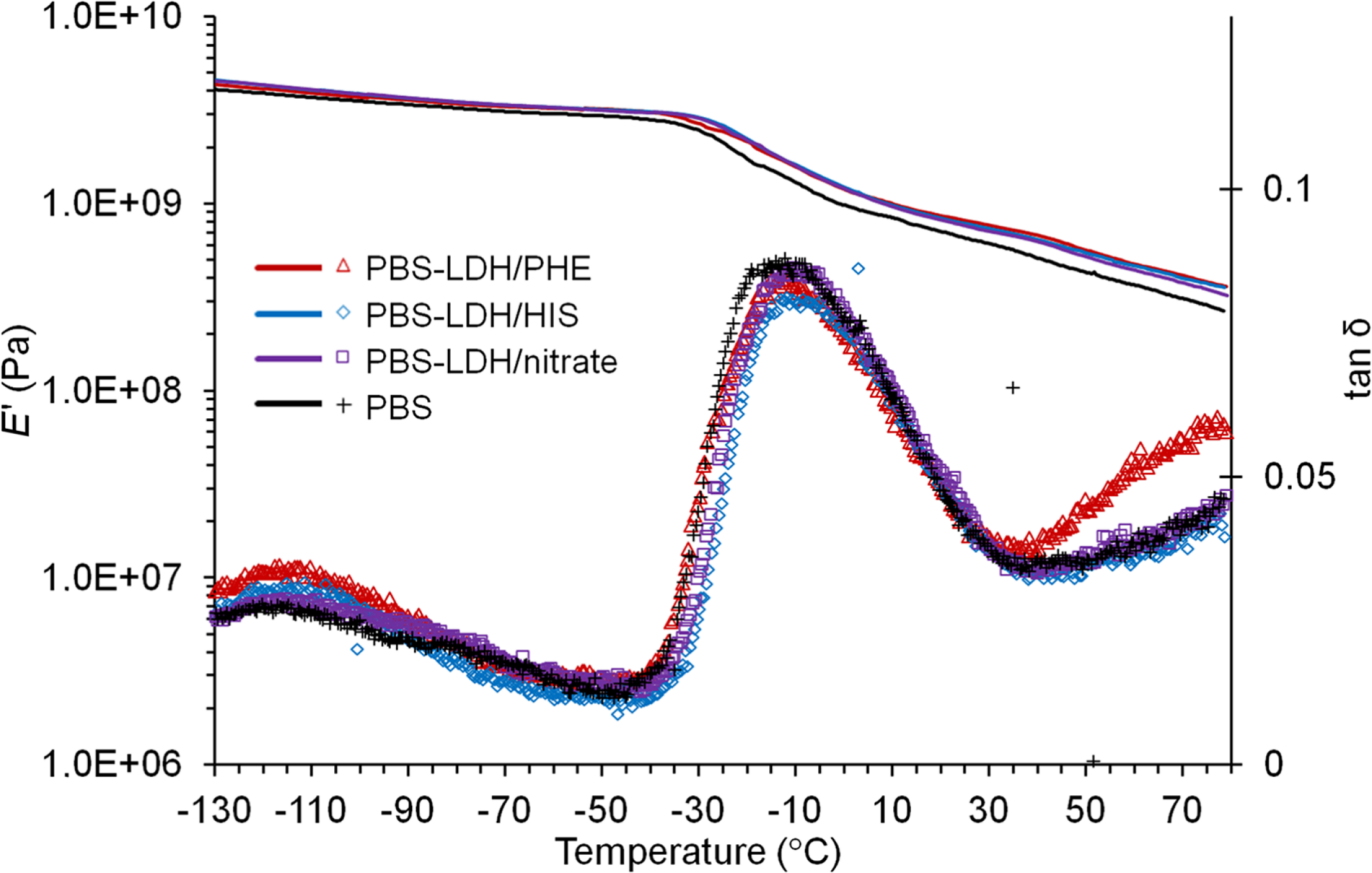
*E*’ and tan δ as a function of temperature for PBS and PBS nanocomposites with Mg_2_Al LDHs.

The temperature and frequency-dependent mechanical relaxation data for the composites were recorded via the storage modulus (*E*′) and tan δ, which is the ratio of the loss modulus to the storage modulus (Figure 8). With respect to PBS, the composites present moderate enhancement in the storage modulus *E*’ over almost the entire temperature range, quantifiable as 6–12% and 17–26% from low temperature (−130 °C) up to RT, respectively. More detailed, at room temperature, the increase in *E*’ is 26% for PBS–LDH/PHE, 20% for PBS–LDH/HIS and 17% for PBS–LDH/nitrate. The larger storage modulus with respect to PBS for such composites indicates a mechanical reinforcement of the matrix due to a better interfacial interaction filler/polymer achieved. Such results are consistent with rheology experiments. For all samples, the glass transition temperature is recognized by the large decrease in the storage modulus and by the corresponding peak maximum in tan δ. The values extrapolated (−15 °C for PBS, −10 °C for PBS–LDH/PHE, −9 °C for PBS–LDH/nitrate and PBS–LDH/HIS) highlight a slight increase with respect to the homopolymer, and therefore, the filler can be assumed to affect the mobility of the chains as a nucleating agent.

### Photostability of PBS nanocomposites

**Figure 9:**
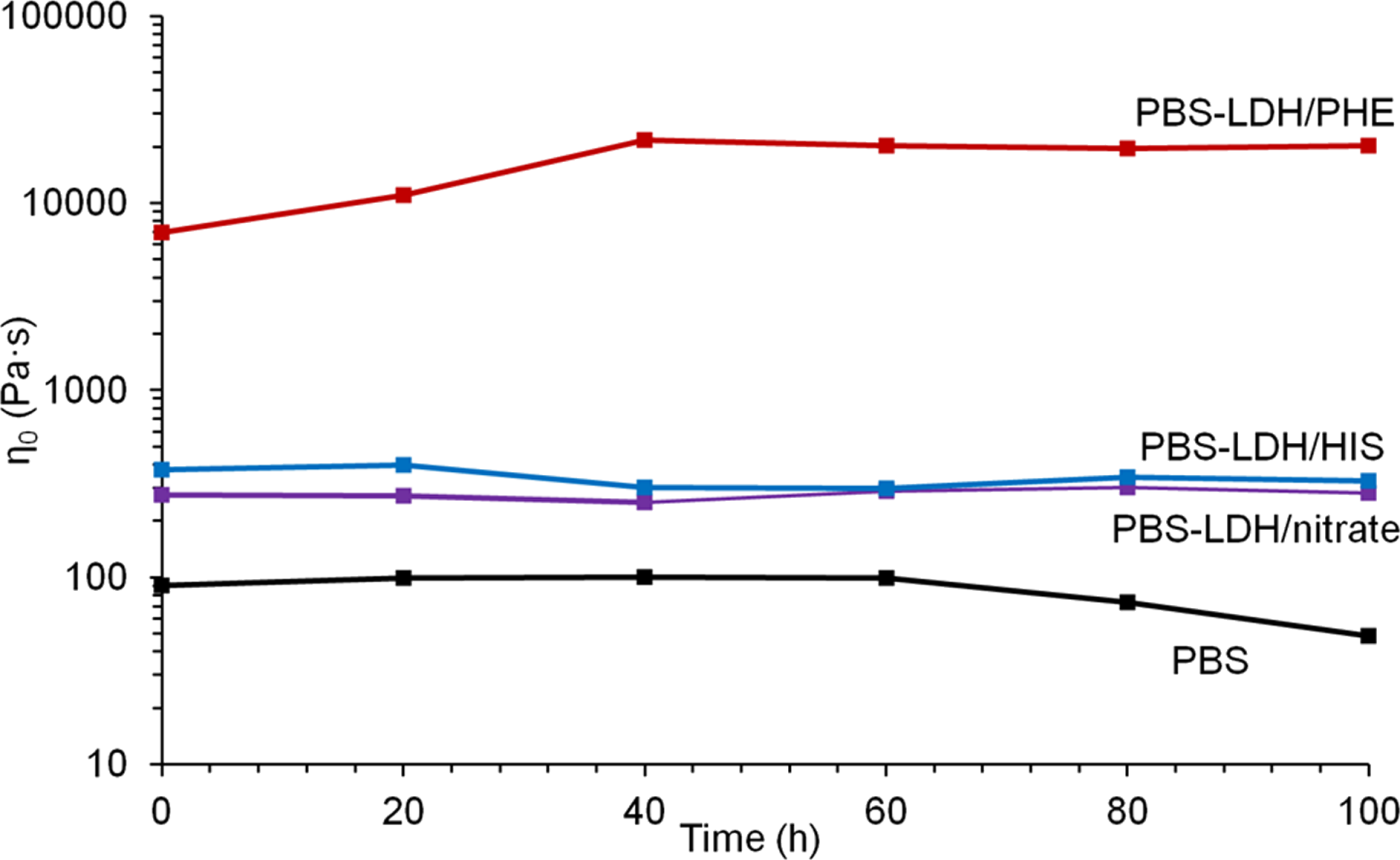
Evolution of zero-shear viscosity (in logarithmic scale) vs time for PBS and PBS nanocomposites with Mg_2_Al LDH during photodegradation tests at 60 °C.

Because all synthesized LDHs show absorption in the UV range (Figure 5), they should be more or less efficient as UV stabilizers. PBS and PBS nanocomposites with Mg_2_Al LDHs were exposed to UV irradiation in an accelerated ageing chamber under aerobic conditions at 60 °C for 100 h. The variation in Newtonian viscosity η_0_ as a function of time is presented in Figure 9. The filler-free PBS sample shows UV resistance for the first 60 h, which then begins to degrade fairly quickly. All three examined Mg_2_Al samples seem to act as UV stabilizers for the examined time period and the small fluctuations of composites with LDH/nitrate and LDH/HIS can result from the heterogeneity of the measured samples. In the case of the PBS nanocomposite with LDH/PHE, which shows the highest extending effect, a further increase in the Newtonian viscosity is observed, probably due to crosslinking reactions. The crosslinking phenomenon in the presence of LDH modified with amino acids has been previously observed and is described in the literature with reference to tryptophan [17].

Up to now, no satisfactory proof was gathered to understand the better characteristics of PHE. Indeed the low content of organic molecules tethered to LDH (≈2 wt % considering the LDH mass loading and respective to LDH:PHE ration inside the hybrid filler) and the overlap of PBS impede any classical spectroscopic characterization. Nevertheless, considering the tethered molecular backbone of PHE as well as the Cole–Cole response indicative of a chain-extension effect rather than a gel-like transition, this may indicate the presence of a jamming structure due to mobility hindrance of PBS chains as observed for other LDH composites with polystyrene [27] rather that a possible covalent or hydrogen-type bonding between tethered PHE and PBS.

The worst behaviour in terms of UV stability for Mg_2_Al/HIS (even if the UV absorption is similar between the two fillers (Figure 5)) may be explained by the strong networking of Mg_2_Al/PHE and also to some degradative effects of Mg_2_Al/HIS likely due to the presence of impurities (as evidenced by XRD), which may be affected under UV radiation of the polymer chains.

**Figure 10:**
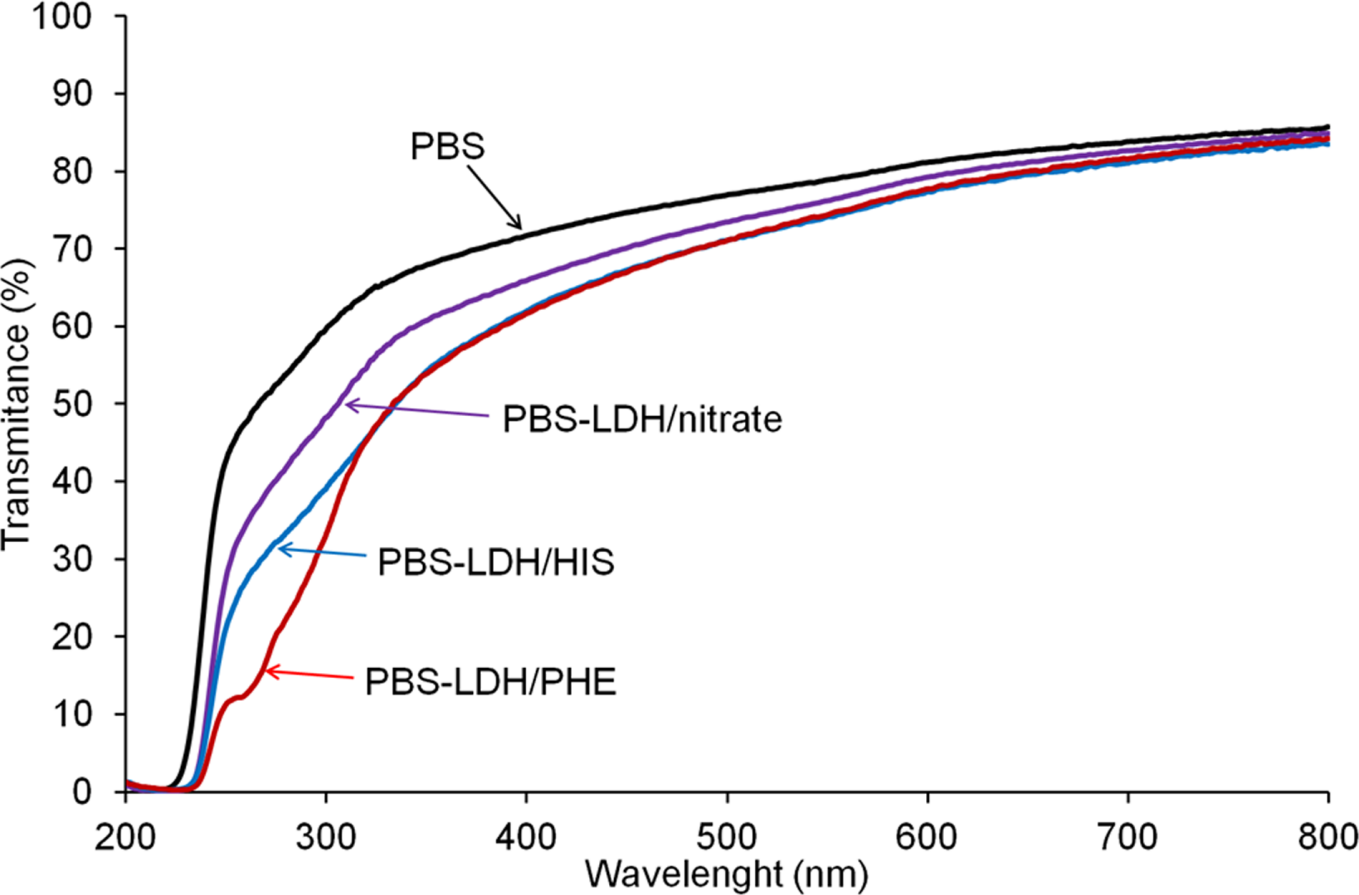
UV–vis transmittance spectra of PBS and PBS nanocomposites with Mg_2_Al LDH fillers.

UV–vis transmittance spectra of PBS and the nanocomposites with 5 wt % of Mg_2_Al LDHs are presented in Figure 10. The largest decrease in transmittance is observed for PBS–LDH/PHE, then for PBS–LDH/HIS. As mentioned for the hybrid LDH materials, the absorption band can be caused by the cycle phenyl or imidazole acting as a chromophore. Also, PBS with Mg_2_Al/nitrate LDH shows lower transmittance, as described above. The evolution of the UV–vis transmittance of PBS and PBS nanocomposites was measured during photodegradation at 60 °C (Supporting Information File 1, Figure S5). The transmittance spectra for PBS are not modified during the entire period of irradiation. In the case of PBS nanocomposites with LDH/nitrate and LDH/HIS, the transmittance decreases during the first 20 h and then stabilizes. When the PBS nanocomposite with LDH/PHE is irradiated, the transmittance decreases during 40 h and then increases. This phenomenon may be caused by the transformation of amino acid molecules in the LDH structure or their interaction with the polymer matrix.

**Figure 11:**
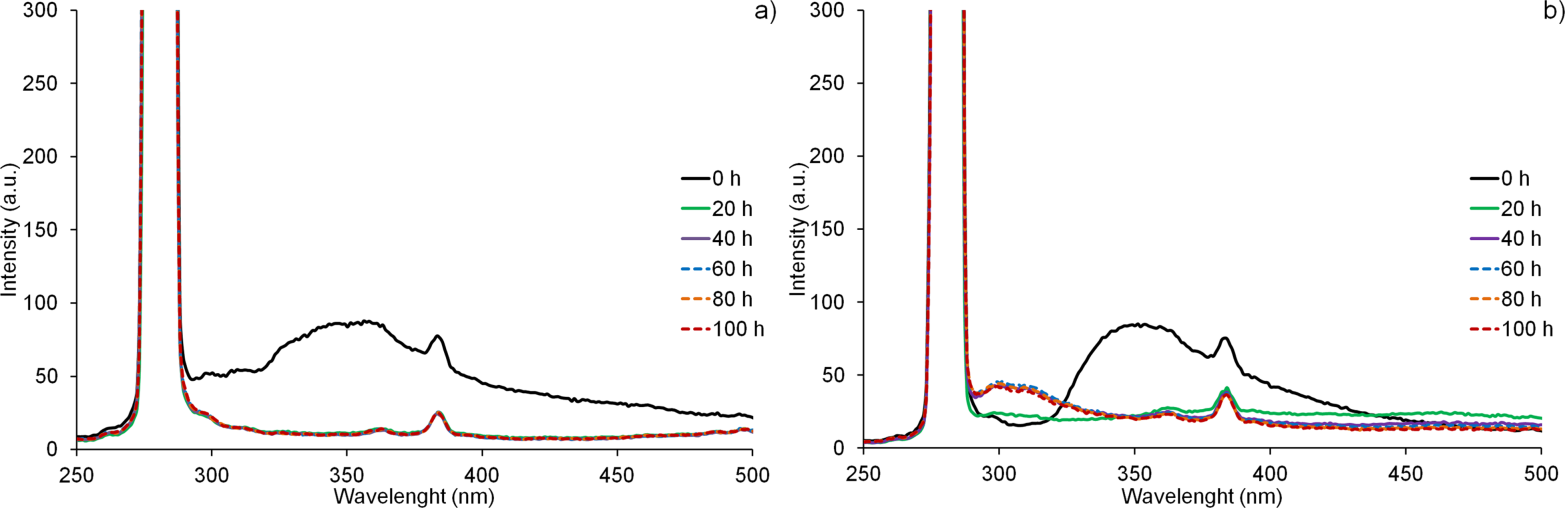
Fluorescence spectra of PBS nanocomposites with Mg_2_Al LDH fillers, during photodegradation tests at 60 °C; (a) PBS–LDH/HIS and (b) PBS–LDH/PHE. The excitation wavelength was 280 nm.

Similar trends have been also registered using fluorescence spectroscopy (Figure 11). The intense emission band centred at 350 nm can be observed for interlayered HIS and PHE (the small peak at 382 nm is instrumental noise). This band disappears after 20–40 h, which is in agreement with the decrease in the UV transmittance described above. In the case of the PBS nanocomposite with LDH/PHE, the emission band shifts towards lower wavelengths, at about 300 nm, which is characteristic for pristine amino acid.

A similar phenomenon was observed for the PBS composite with Zn_2_Al/TYR LDH and is described in the literature [15]. It can be explained by the delamination of the LDH platelets and the presence of LDH filler in a different environment after UV irradiation.

## Conclusion

Two amino acids, L-phenylalanine and L-histidine, have been interleaved into Mg_2_Al layered double hydroxides and subsequently dispersed in PBS by melt blending. The XRD analysis indicated the presence of nitrate phase in the LDH structure, which can be explained by the large excess of nitrate anions in the reaction medium during co-precipitation. For this reason, the LDH framework with nitrate anions was also synthesized and applied for PBS as a reference.

The best results were obtained in the case of the LDH/PHE filler. By the use of melt rheology, an outstanding chain-extending effect was observed, with an increase of the Newtonian zero-shear viscosity of almost 90 times in comparison to filler-free PBS samples.

Moreover, the synthesized organo-modified LDHs were found to be effective as UV-stabilizers since they successfully prevent the chain scission reactions which usually occur during photo-ageing of PBS. Especially in the case of LDH/PHE, the UV-stabilizing effect is quite sustainable over time, thus making such a filler composition a good alternative in the design of polymer composites with these specific properties. These properties are important from the point of view of processability as well as for their prolonged shelf life and extended use.

## Supporting Information

File 1 Additional experimental data and experimental schemes.
