# Supplementary material for: Outstanding chain-extension effect and high UV resistance of polybutylene succinate containing amino-acid-modified layered double hydroxides
Source: Beilstein J Nanotechnol. 2019 Mar 12;10:684–95. doi: 10.3762/bjnano.10.68 (PMC6423599; doi:10.3762/bjnano.10.68)
Supplement: File 1 — Additional experimental data and experimental schemes. [file Beilstein_J_Nanotechnol-10-684-s001.pdf]

BEILSTEIN JOURNAL OF NANOTECHNOLOGY

Supporting Information

for

Outstanding chain-extension effect and high UV resistance of polybutylene succinate containing amino-acid-modified layered double hydroxides

Adam A. Marek, Vincent Verney, Christine Taviot-Gueho, Grazia Totaro, Laura Sisti, Annamaria Celli and Fabrice Leroux

Beilstein J. Nanotechnol. **2019,** *10,* 684–695. doi:10.3762/bjnano.10.68

Additional experimental data and experimental schemes

License and Terms: This is a supporting information file under the terms of the Creative Commons Attribution License (http://creativecommons.org/licenses/by/4.0). Please note that the reuse, redistribution and reproduction in particular requires that the authors and source are credited.
The license is subject to the *Beilstein Journal of Nanotechnology* terms and conditions: (https://www.beilstein-journals.org/bjnano)

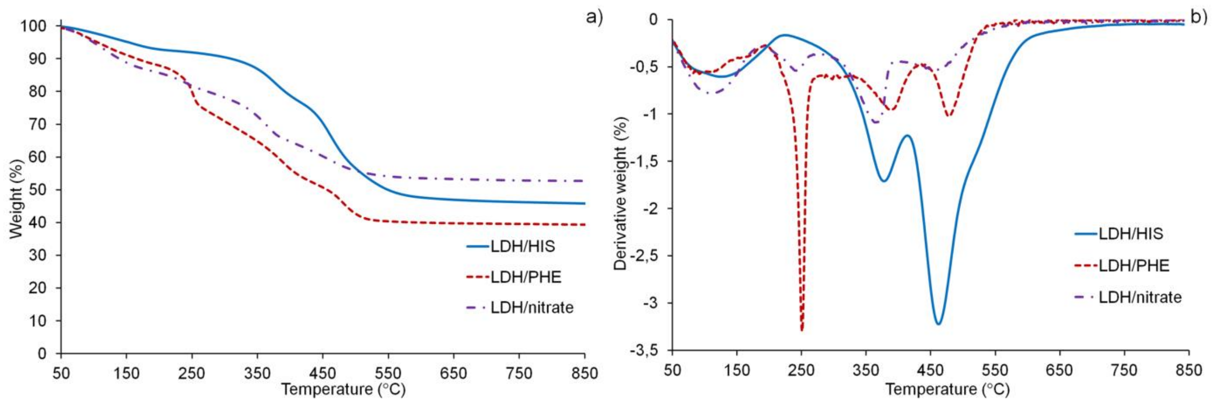

Figure S1: TGA (a) and DTGA (b) traces of Mg_2Al LDHs, under air flow

The procedure for calculation of experimental chemical compositions on example of LDH/HIS

The general formula of synthesized LDH with molecular weight $\mathbf{M_{RT}}$ is:

$$\mathbf{[Mg_2Al(OH)_6]A^-_{(1-x)}NO_3^-{}_x\ nH_2O}.$$

XRD analysis excludes the presence of carbonates but confirms nitrate anions in interlayers space.

Data:	Anion HIS^-	$M_w = 154.15$ g·mol^{-1}
	Anion NO_3^-	$M_w = 62.00$ g·mol^{-1}
	Mg_2AlOH_6	$M_w = 177.64$ g·mol^{-1}
	$Mg_2AlO_{7/2}$	$M_w = 131.58$ g·mol^{-1}
	Mass of the residue at 800 °C ($Mg_2AlO_{7/2}$)	= 55 wt %

The calculation is based on TGA traces.

$$M_{RT}(\text{LDH/HIS}) = 131.58/(1–0.55) = 292.4 \text{ g mol}^{-1}$$

The water molecule content “n” is calculated from the mass loss after the first relative minimum on the derivative curve ≈ 170°C. In the case of LDH/HIS The mass loss of H_2O is 7 wt %.

Mass $H_2O = M_{RT} \cdot \%H_2O = 292.4 \cdot 0.07 = 20.47$ g » n $H_2O = 20.47/18.02 = 1.14$ mol

Next, the amount of HIS and nitrate anions are calculated:

154.15·(1–x) + 62·x = 292.4 – 177.64 – 1.14·18.02 » x = 0.65

The chemical composition for LDH/HIS is **$[Mg_2Al(OH)_6](HIS^-)_{0.35}(NO_3^-)_{0.65}$ 1.14H_2O**.

Similar calculations have been done for LDH/PHE, where:

$$M_{RT}(\text{LDH/PHE}) = 131.58/(1–0.62) = 346.26 \text{ g mol}^{-1}$$
and H_2O = 11.5 wt % » **n** H_2O = 2.21 mol

In the case of LDH/nitrate: $M_{RT}(\text{LDH/nitrate}) = 131.58/(1–0.48) = 253.04 \text{ g mol}^{-1}$
because only nitrate anions are present in the interlayered space (FTIR, XRD analysis), in this case, H_2O was calculated from the difference:

$$\text{Mass of } H_2O = M_{RT}(\text{LDH/nitrate}) – Mg_2Al(OH)_6 – NO_3^- = 253.04 – 177.58 – 62 = 13.46 \text{ g}$$
» » **n** H_2O = 13.46/18.02 = 0.75 mol

It means that at 200 °C there is not only loss of water but also deshydroxylation.

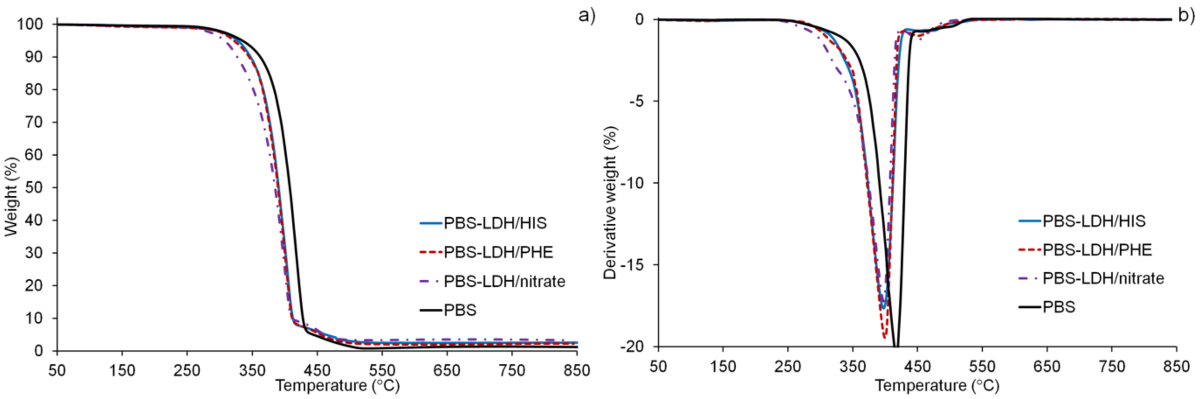

Figure S2: TGA (a) and DTGA (b) traces of PBS and PBS nanocomposites with Mg_2Al LDH fillers, under air flow.

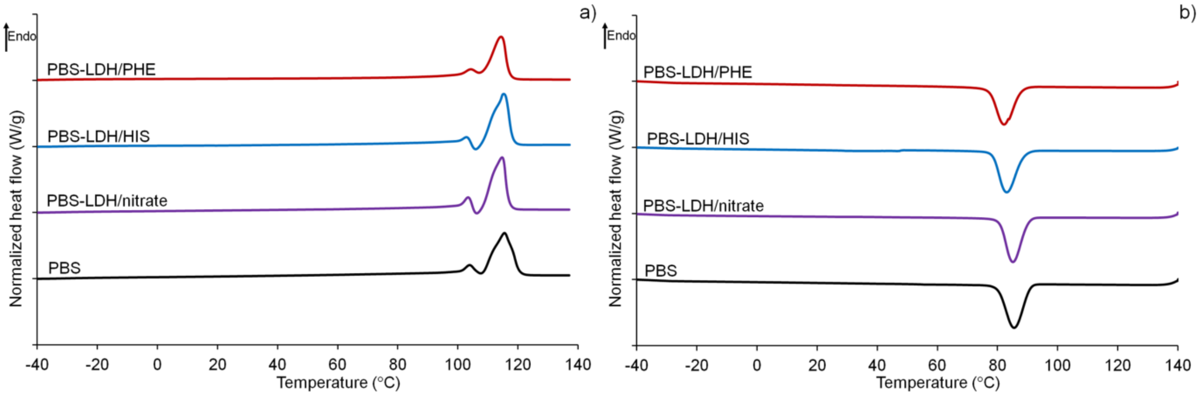

Figure S3: DSC traces of PBS and PBS nanocomposites with Mg_2Al LDH fillers: (a) heating scans and (b) cooling scans.

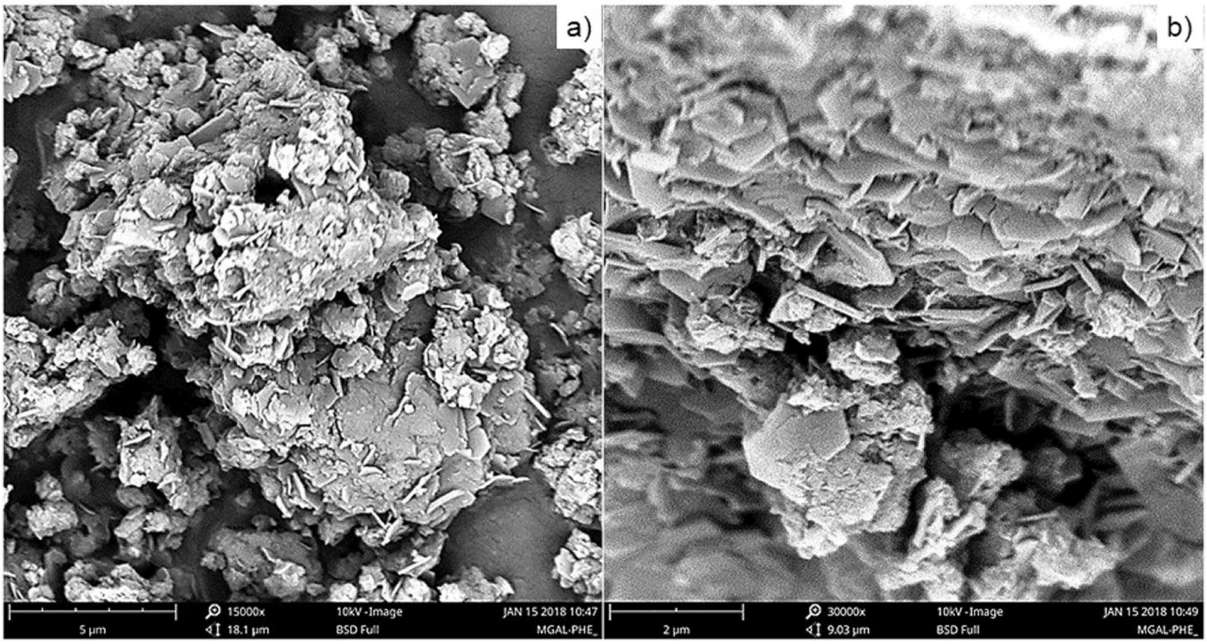

Figure S4: SEM micrographs of Mg_2Al LDH with phenylalanine: (a) zoom 15000x, (b) zoom 30000x.

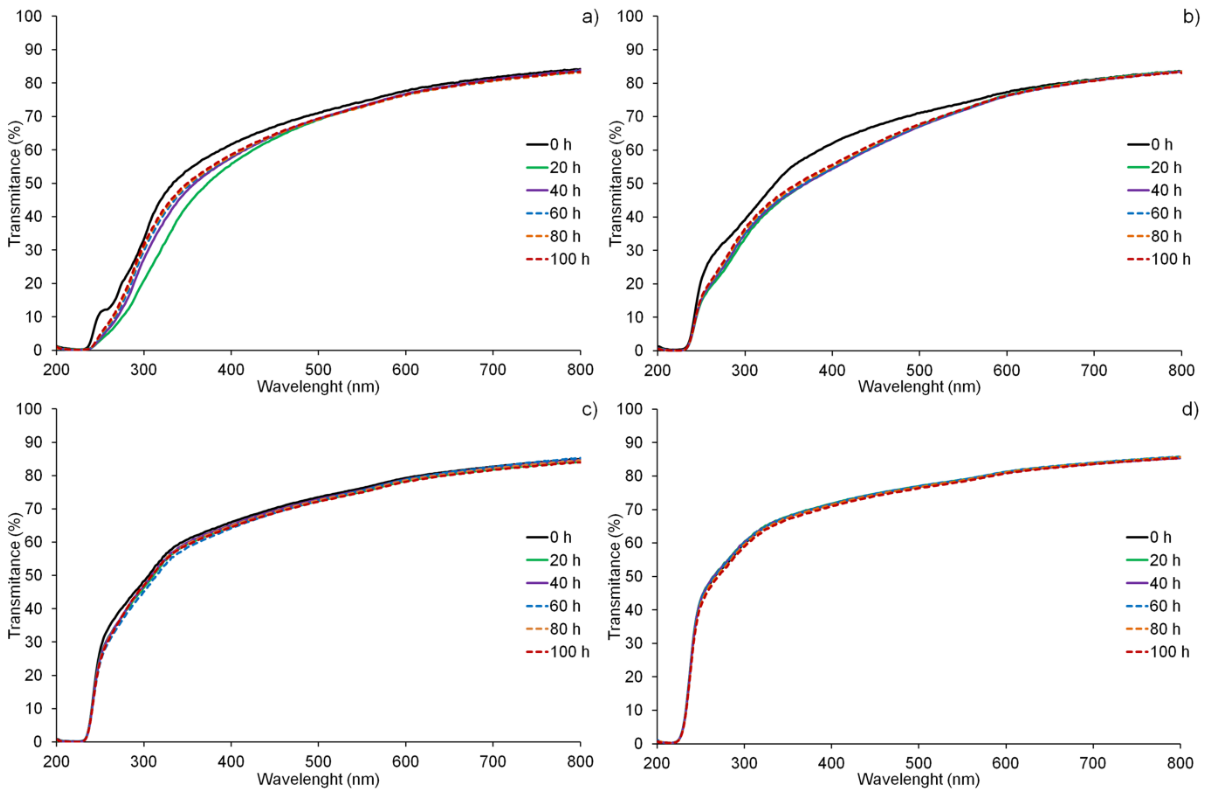

Figure S5: UV–vis transmittance spectra of PBS and PBS nanocomposites with Mg_2Al LDH fillers, during photodegradtion at 60 °C; (a) LDH/PHE, (b) LDH/HIS, (c) LDH/nitrate and (d) PBS.
